# Screening for a practical method to monitor the status of patients with metastatic bladder cancer at the circulating cell-gene level

**DOI:** 10.1038/s41598-023-46977-1

**Published:** 2023-11-09

**Authors:** Ryota Ogura, Saya Ito, Takashi Ueda, Yusuke Gabata, Satoshi Sako, Yuta Inoue, Takeshi Yamada, Hirotaka Konishi, Atsuko Fujihara, Osamu Ukimura

**Affiliations:** 1 Department of Urology, Graduate School of Medical Science, Kyoto Prefectural University of Medicine, Kyoto, Kyoto 602-8566 Japan; 2 Division of Digestive Surgery, Department of Surgery, Kyoto Prefectural University of Medicine, Kyoto, Kyoto Japan

**Keywords:** Bladder cancer, Cancer therapy

## Abstract

Identifying a novel method to monitor metastatic bladder cancer status at the cell-gene level could lead to earlier appropriate therapeutic intervention and better outcomes. In this study, we evaluated a practical method to monitor the cancer status at the circulating cell-gene level before and after treatment in fourteen patients with metastatic bladder cancer who were indicated for systemic drug therapy. Patients were assessed via imaging before and after drug treatment, and cell-free DNA (cfDNA) analysis was performed to detect three parameters: cfDNA level, *ERRB2* gene copy numbers, and telomerase reverse transcriptase (*TERT*) gene mutations. We hypothesized that decreased cfDNA levels, a normal copy number of ERB-B2 receptor tyrosine kinase 2 (*ERBB2*), and the absence of the *TERT* C228T mutation indicate cancer suppression. We found that a > 1.8-fold increase in cfDNA levels, increased copy number of *ERBB2*, or the existence of the *TERT* C228T mutation indicated disease progression. Stable cfDNA levels, normal *ERBB2* copy number, and the absence of *TERT* C228T mutations indicate a stable cancer status. Collectively, our results show that the combination of cfDNA concentration, *TERT* mutation, and *ERBB2* copy number may be useful for determining the efficacy of drug therapy in patients with metastatic bladder cancer.

## Introduction

Urothelial carcinoma is a malignant disease arising from urothelial cells in the urinary tract, including the bladder. According to a global survey in 2012, the number of patients newly diagnosed with urothelial carcinoma was 430,000, and 165,000 deaths were reported^1^. While localized bladder cancer has a favorable outcome, with a 5-year survival rate of 77%, metastatic bladder cancer (mBC) has a poor prognosis, with a 5-year survival rate of only 5.5%^2^. The standard therapy for mBC is cisplatin-based chemotherapy without surgery; however, its efficacy is insufficient^3^. In recent years, new drugs such as immune checkpoint inhibitors (ICIs) and enfortumab vedotin (EV) have emerged and are recommended for some patients^4,5^. However, a method to monitor cancer status at the circulating cell-gene level for mBC has not yet been established, and imaging for the macroscopic observation of cancer status is still currently used to determine drug efficacy and therapeutic intervention. Hence, identifying a novel method to monitor mBC status at the circulating cell-gene level could lead to earlier appropriate therapeutic intervention and better outcomes.

Cell-free DNA (cfDNA) was first identified by Mandel and Metais in 1948^6^. In 1977, Leon et al. reported that cfDNA levels in the blood of patients with various types of metastatic cancers were higher than those in patients without metastases^7^. Recently, Papadimitriou et al. reported that elevated preoperative cfDNA levels in localized muscle-invasive bladder cancer (MIBC) were associated with a high risk of short-term postoperative recurrence and poor prognosis^8^. Furthermore, an increase in cfDNA levels after surgery for localized MIBC has been reported to be associated with the disease^9^. Accordingly, observing cfDNA levels before and after drug therapy for mBC could be useful for monitoring cancer status.

Tumor-derived cell-free DNA (ctDNA), which is cfDNA released from cancer cells, is present in the peripheral blood of patients with cancer through apoptosis, necrosis, and cytolysis^10^. The concordance rate of DNA aberrations detected in ctDNA with DNA aberrations from contemporaneously sampled tumor tissues has been very high^11^. In urothelial carcinoma, genomic profiles analyzed via next-generation sequencing of cfDNA samples have been reported to be very similar to those of ctDNA and tissue specimens^12^. Christensen et al. reported that ctDNA analysis via ultra-deep sequencing in patients with localized advanced bladder cancer showed that ctDNA dynamics during drug therapy were associated with recurrence in patients before or during treatment^13^.

Because cfDNA and ctDNA have the advantages of being less invasive, more repeatable, and could overcome tumor heterogeneity compared with tissue biopsy, cfDNA and ctDNA have attracted attention as promising biomarkers for various cancer types^14^. Although a comprehensive analysis of ctDNA via whole-genome sequencing of cfDNA is ideal from the perspective of precision medicine, it is not yet practical for current clinical use. The quantitation of cfDNA levels in patients is more practical in terms of its applicability to a broad range of patients and is both cost-effective and time-effective. However, it can be affected by other factors, such as inflammation.

In this study, we prospectively collected cfDNA samples from mBC patients treated with various drug therapies. We explored tools for monitoring the status of mBC in clinical practice by quantifying cfDNA levels and detecting ctDNA as an indicator of genetic mutations in cfDNA, including the most common mutations in BC.

## Results

### Patient characteristics

Fourteen patients with mBC were prospectively enrolled in this study (Table 1). The median age was 77 years (range: 55–86 years). Total cystectomy was performed in six patients (43%). Twelve patients were male and two was female. All cases included urothelial carcinoma. Five patients (36%) had a variant histology. The sites of metastasis were the regional lymph nodes in eleven cases (79%), distant lymph nodes in four cases (29%), and distant organs in three cases (21%).

**Table 1:**
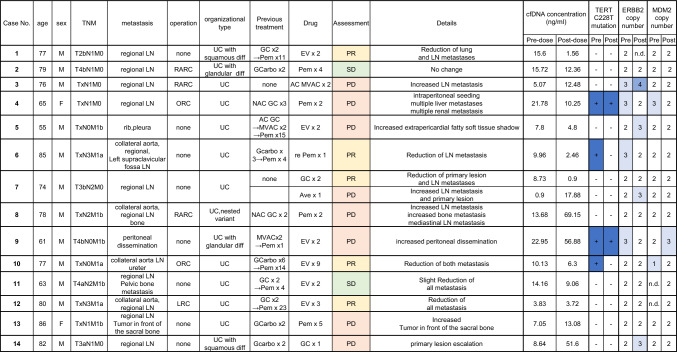
**Clinical characteristics of the patients and drug contents.**

Information on the cfDNA extracted from each patient (cfDNA concentration and genetic mutations) is shown on the right side of the table.

*LN* lymph node, *RARC* robot-assisted radical cystectomy, *ORC* open radical cystectomy, *UC* urothelial carcinoma, *diff* differentiation, *GC* gemcitabine and cisplatin, *Pem* pembrolizumab, *GCarbo* gemcitabine and carboplatin, *NAC* neoadjuvant chemotherapy, *AC* adjuvant chemotherapy, *MVAC* methotrexate and vinblastine and adriamycin and cisplatin, *EV* enfortumab vedotin, *re Pem* pembrolizumab rechallenge, *Ave* avelumab, *PR* partial response, *SD* stable disease, *PD* progressive disease, −, negative; + , positive; *n.d.* not detected.

Treatment efficacy was determined as PR (partial response) in five cases, SD (stable disease) in two case, and PD (progression disease) in eight cases. Because case 7 was assessed before and after GC (Gemcitabine/Cisplatin) therapy and avelumab treatment, PR and PD decisions were made. Drug therapies in the PR group included GC (case 7), pembrolizumab (case 6), and EV (case 1, 10, 12). Drug therapy in the SD group included pembrolizumab (case 2), pembrolizumab (case 11). Drug therapies in the PD group included MVAC (Methotrexate/Vinblastine /Adriamycin/Cisplatin) (case 3), pembrolizumab (cases 4, 8, 13), avelumab (case 7), EV (case 5, 9), and GC (case 14).

### Observation of ctDNA in patients with mBC as a possible biomarker to monitor therapeutic efficacy

**Figure 1:**
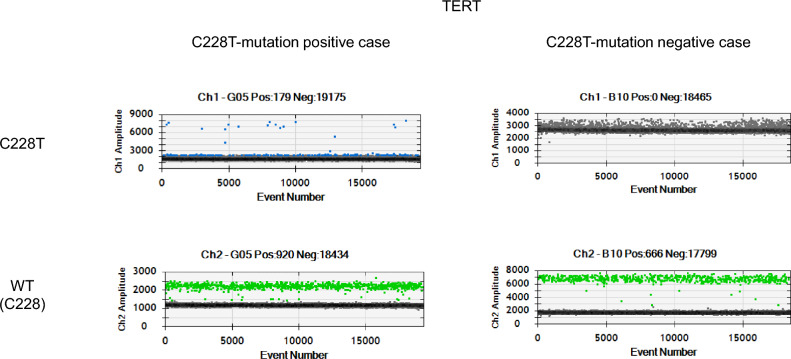
Cases of C228T point mutations in the *TERT* gene as detected using ddPCR. The upper panel shows the quantitative results of ddPCR using primers to detect the C228T mutant *TERT* gene (blue dots), while the lower panel shows the wild-type (green dots). The left panels show the results of cfDNA analysis from the samples of case 4 that were positive for the C228T mutation, and the right panels show the detection of the wild-type *TERT* gene.

Genetic mutations in the telomerase reverse transcriptase (*TERT*) gene and phosphatidylinositol-4,5-bisphosphate 3-kinase catalytic subunit alpha (*PIK3CA*) gene are prevalent in bladder cancer. *TERT* gene mutations in bladder cancer tissue were observed in approximately 67% of cases and *PIK3CA* gene mutations in approximately 20%^16,17^. Szabados et al. found no postoperative recurrence in patients with undetectable ctDNA levels before and after preoperative atezolizumab therapy, indicating that ctDNA may be a useful prognostic factor^18^. Therefore, we investigated the usefulness of *TERT* and *PIK3CA* mutations as blood biomarkers for bladder cancer. The C228T and C250T mutations in the *TERT* gene and the E545K mutation in the *PIK3CA* gene were tested in the first specimen collected in all cases. A pretreatment case 4 with positive cfDNA and one case with negative cfDNA are shown. (Fig. 1). In case 6 and 10, C228T mutations in *TERT* were detected before treatment, but it disappeared after treatment. Although ctDNA could be an indicator of PR and PD, mutations were only detected in case 4, 6, 9, 10 (1/8, 29% Table S1). We concluded that the three mutations in the *TERT* and *PIK3CA* genes were too low to monitor patients with mBC in clinical practice.

### Quantification of cfDNA in patients with mBC is a possible biomarker to monitor therapeutic efficacy

**Figure 2:**
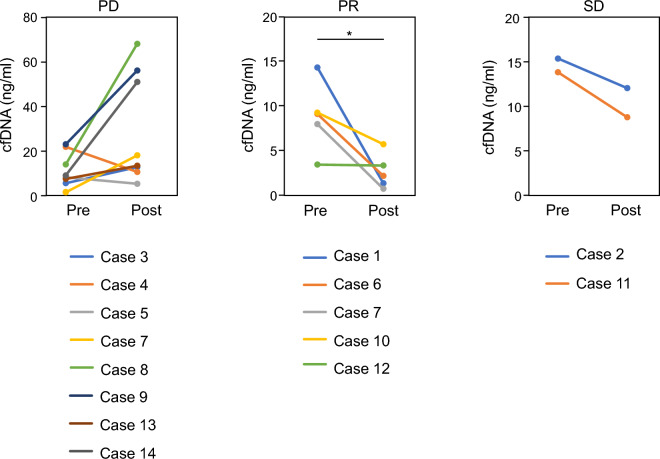
Quantification of cfDNA levels before and after treatment. Changes in cfDNA levels before and after treatment are shown for the PD, PR, and SD groups. In the PR group, cfDNA levels were clearly reduced after treatment (p < 0.05). In the PD group, cfDNA levels tended to increase in the chemotherapy group.

**Figure 3:**
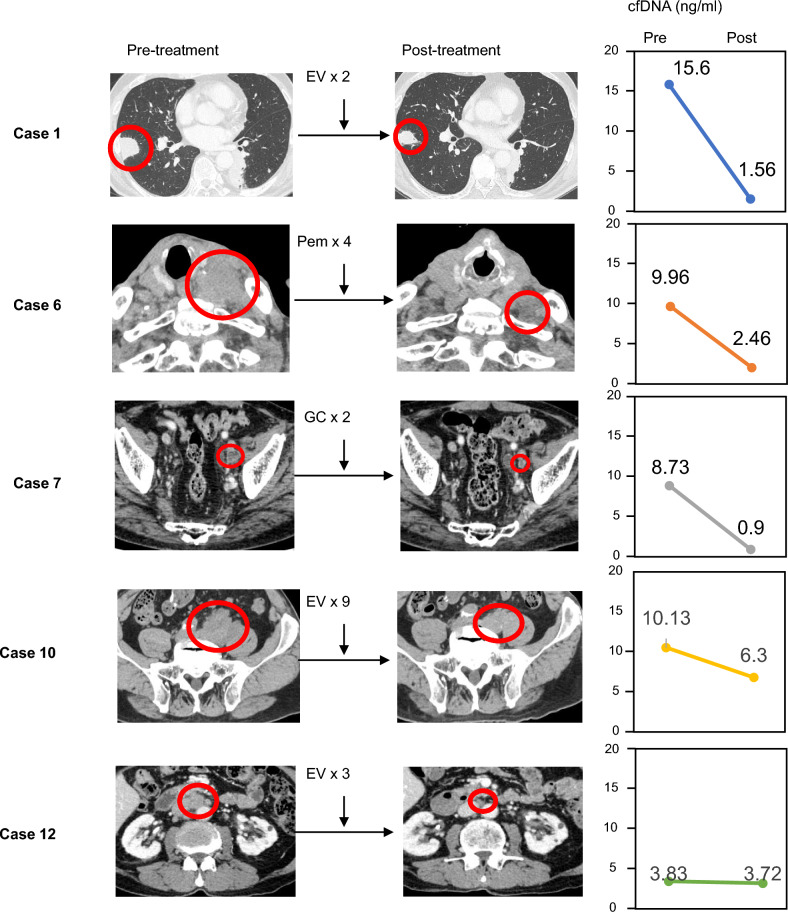
CT images and cfDNA levels in the PR group. All regimens regressed cancer (CT images, left panels) and reduced cfDNA levels (cfDNA levels, right graphs) post-treatment compared to that pre-treatment.

Next, we examined the possibility that fluctuations in cfDNA levels could be useful biomarkers. The normal cfDNA concentration is 10 ng/mL^19,20^. Using blood samples, we validated the cfDNA levels in healthy volunteers under our experimental conditions. The median normal cfDNA concentration under our experimental conditions was 3.78 ng/mL (Table S2). In the PR group, a significant decrease in cfDNA levels was observed before and after treatment (Figs. 2 and 3) (p < 0.05). In the SD group, cfDNA levels were not significantly different before and after treatment (Fig. 2 and Fig. S1). In the PD group, although cfDNA levels tended to increase in the chemotherapy group, no significant difference was observed (Fig. 2) (p = 0.0605). From the above results, we observed an association between cfDNA levels and the outcomes observed radiographically in each case in the PR group.

In case 1, EV was administered for 2 courses and the cfDNA level decreased from 15.6 to 1.56 ng/mL at PR determination. In case 6, pembrolizumab was administered for 4 courses and the cfDNA level decreased from 9.96 to 2.46 ng/mL at PR determination. In case 7, GC therapy was performed for 2 courses and the cfDNA level decreased from 8.73 to 0.9 ng/mL at PR determination. In case 10, EV was administered for nine courses and the cfDNA level decreased from 10.13 to 6.3 ng/mL at PR determination. In case 12, EV was administered for 3 courses and the cfDNA level decreased from 3.83 to 3.72 ng/mL at PR determination (Fig. 3). Pre- and post-dose cfDNA levels are shown in Table 1.

### An increased copy number of the *ERBB2* gene in the cfDNA indicates cancer progression

In the PD group, cfDNA levels in cases 3, 7, 8, 9, 13, 14 increased, but those in the other patients did not. Therefore, we examined whether indicators other than cfDNA could be used as biomarkers for mBC. In bladder cancer, common genetic mutations include copy number changes in the *MDM2* and *ERBB2* genes^21^. Soave et al. showed that *ERBB2* copy number variation was associated with aggressive tumor characteristics, using DNA from tissues and serum cfDNA of patients with bladder cancer^22^.

**Figure 4:**
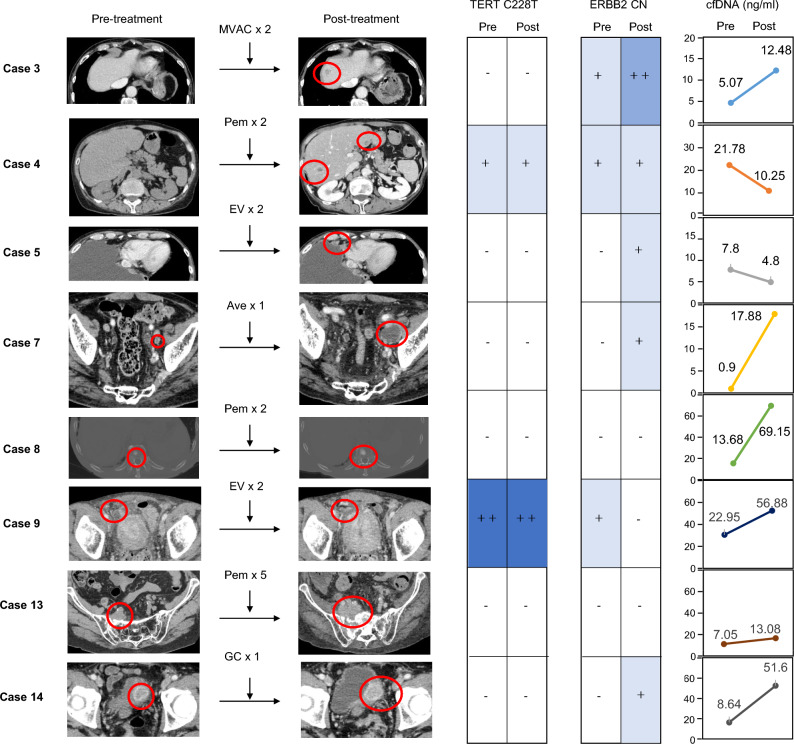
CT images and genetic mutations in the cfDNA of patients in the PD group. All regimens progressed disease (CT images, left panels), while the changes in cfDNA levels were varied (cfDNA levels, right graphs). In case 4, both a C228T mutation in the *TERT* gene and copy number gain in *ERBB2* were detected. In cases 3, 5, 7 and 14, the copy number of the *ERBB2* gene increased after treatment compared to that before treatment.

Based on these reports, we hypothesized that copy number changes in *MDM2* and *ERBB2* in the cfDNA could be used as biomarkers for mBC. We then evaluated the copy numbers of the *MDM2* and *ERBB2* genes in the cfDNA, as well as the treatment decision to determine PR or PD. In the PR group, the copy number of *MDM2* in the cfDNA was below normal or undetectable. In the PD group, the copy number of *MDM2* in cfDNA increased only in case 9 (Fig. 4). In the PR group, one case could not be measured after treatment, and one case had a normal *ERBB2* copy number in the cfDNA after treatment. In the PD group, four cases had an increased *ERBB2* copy number in their cfDNA after treatment (Fig. 4).

## Discussion

In this study, we screened for a practical method to monitor cancer status at the circulating cell-gene level using blood samples from patients with mBC. We determined three parameters in our cfDNA analysis: cfDNA concentration, *TERT* gene mutations (C228T), and *ERBB2* gene copy numbers. From our results, we hypothesized the following combination algorithm: a decrease in cfDNA level, normal *ERBB2* copy number, and absence of *TERT* C228T mutations after drug therapy indicate the suppression of cancer (PR). A more than 1.8-fold increase in cfDNA level, increased *ERBB2* copy number, or existence of the *TERT* C228T mutation after drug therapy indicated disease progression (PD). Stable cfDNA levels, normal *ERBB2* copy number, and absence of the *TERT* C228T mutation after drug therapy indicated stable disease (SD) (Fig. S2).

In clinical practice, the combination of specific cfDNA targets presented in this study may be useful as an adjunct diagnostic tool for the early detection of metastasis. Currently, imaging is used to determine the efficacy of drug therapy. However, a PD decision is not made until tumor growth or metastasis is grossly confirmed. The detection of micrometastases and earlier appropriate intervention may prolong survival.

Recently, ctDNA research has progressed significantly. Christensen reported that a ctDNA-based analysis of recurrence after total cystectomy showed a median lead time beyond 96 days of imaging diagnosis and that ctDNA dynamics during drug therapy were associated with recurrence in ctDNA-positive cases before or during treatment^13^. However, the clinical use of ctDNAs is challenging. Minute amounts of ctDNA are difficult to analyze as typical detection methods still have low sensitivity. Another disadvantage of this method is that gene mutations vary from tissue to tissue, and individual analyses are required for each patient, which could be costly and time-consuming. Furthermore, it has recently become clear that even normal hematopoietic cells accumulate genetic mutations in response to external stimuli (clonal hematopoiesis of indeterminate potential; CHIP)^23^. There have been reports of cases where the identified genetic mutation was a CHIP mutation. Moreover, differentiating CHIP from ctDNA is challenging. In addition, urinary cf/ctDNA (ucfDNA) has been studied in recent years. However, some reports have indicated that a liquid biopsy of urine samples using a gene panel is not effective in diagnosing bladder cancer^24^. In the future, it will be necessary to identify additional factors using comprehensive ctDNA analysis via whole-genome DNA sequencing of cfDNA using patients’ serum and urine samples.

Our study suggests that the simultaneous monitoring of ctDNA and cfDNA levels in the blood is a useful indicator for cancer diagnosis. In other cancer types, cfDNA levels and the prediction of therapeutic responses have been reported. Chen et al. reported significantly better disease control rates in patients with advanced cell lung cancer who had at least a 20% reduction in cfDNA levels after 6 weeks of treatment^25^. Yu et al. reported that cfDNA levels tended to decrease or change in the PR group and were higher in the SD and PD groups during preoperative chemotherapy for advanced gastric cancer^26^. Hassan et al. reported significantly increased cfDNA levels in a group of patients with breast cancer who experienced recurrence during postoperative follow-up compared to a recurrence-free group^27^. Feng compared cfDNA levels in patients treated with sorafenib for metastatic renal cancer and reported a decrease in cfDNA levels in the PR group, but an increase in the SD and PD groups^28^.

Papadimitriou et al. reported that elevated preoperative cfDNA levels in MIBC were associated with a high risk of short-term postoperative recurrence and poor prognosis^8^. Just as elevated preoperative cfDNA levels are associated with postoperative recurrence, cfDNA levels may also be associated with pathogenesis with respect to pharmacotherapy; however, this has not been extensively reported. However, bladder cancer-related copy number abnormalities have been previously reported. For instance, Millis et al. reported that *ERBB2* is amplified in bladder cancer^29^. Vandekerkhove et al. reported that 95% of patients with advanced bladder cancer with genomic mutations had *TP53*, *RB1*, or *MDM2* mutations and 20% had *ERBB2* amplification^30^. This study suggests that combining cfDNA levels and *ERBB2* copy number in patients might predict the efficacy of drug therapy. A limitation of this study is its small sample size. Furthermore, there was heterogeneity in the groups, as some cases began sampling during drug therapy. In some cases, such as cases 6 and 7, the cfDNA level clearly reflected the disease status; in others, it was not. Because cfDNA levels are likely to be affected by various factors, it is necessary to consider the circumstances under which cfDNA can be collected and quantified. Hence, future studies should be designed on a large number subjects and begin by taking samples from the first drug treatment, and further investigations are needed to determine whether cfDNA levels can be used as biomarkers for clinical applications.

## Methods

### Patients and study design

Fourteen patients with mBC indicated for systemic drug therapy between January 2021 and February 2023 were included. Patients who began specimen collection during their second- or third-line treatment were also included. Treatment consisted of platinum-based chemotherapy [methotrexate, vinblastine, adriamycin, and cisplatin (MVAC); or gemcitabine and cisplatin (GC)], immune checkpoint inhibitors (pembrolizumab and avelumab), and EV. Consent for participation in this study was obtained from all patients.

Imaging was performed approximately every 2–3 months after administration and analyzed separately for partial response (PR), stable disease (SD), and progressive disease (PD). RESIST ver1.1 was used for the imaging criteria. cfDNA levels and imaging results before and after drug treatment were compared.

Healthy subjects were defined as those with no history of malignancy. Ten healthy subjects were also randomly selected.

This study was approved by the Institutional Review Board affiliated with the Kyoto Industrial Health Association and the Kyoto Prefectural University of Medicine (Approval No. ERB-C-1893-1). Informed consent was obtained from all subjects. This work was conducted following the principles of the Declaration of Helsinki.

### cfDNA extraction and quantification

Blood was collected in whole blood collection tubes (Streck Cell-Free DNA BCT^®^ CE) and stored at room temperature for up to two weeks until DNA extraction process^15^. Blood samples were centrifuged at 1600×*g* for 10 min at 4 °C. The plasma was transferred to a new centrifuge tube and centrifuged at 6000×*g* for 30 min at 4 °C. cfDNA was extracted from the plasma using a QIAamp Circulating Nucleic Acid kit^®^ (Qiagen) according to manufacturer’s instructions. Briefly, for 5 mL plasma samples, cfDNA was isolated via proteolytic digestion with Proteinase K and 1.0 μg carrier RNA at 60 °C for 30 min and then incubated with the buffer ACB for 5 min on ice. The lysate was then transferred to a QIAamp Mini column and washed with the buffers ACW1 and ACW2, and ethanol. The purified cfDNAs were then eluted with 30 μL of the buffer AVE and stored at −80 °C until further analysis. The quality of some cfDNA samples was checked using an Agilent 2100 Bioanalyzer Electrophoresis System^®^. cfDNA was quantified using a Qubit4^®^ system (Invitrogen).

### *PIK3CA and TERT* mutation analysis

A ddPCR Mutation Detection Assays (Bio-Rad) was used to detect mutations in *PIK3CA* gene and the promoter region of the *TERT* gene (C228T and C250T). 1 µl of purified cfDNA was used for each ddPCR reaction (Concentrations of each cfDNA are shown in Table 1). For *PIK3CA*, 1 µl of purified cfDNA was mixed with 10 µl of ddPCR Supermix for Probe no dUTP (Bio-Rad), 1 µl of ddPCR Mut Assay, and 8 µl of water in 96 well plate. For *TERTC228T and TERTC250T*, 1 µl of purified cfDNA was mixed with 10 µl of ddPCR Supermix for Probe no dUTP, 1 µl of ddPCR Mut Assay, 1.0 µl of 5 M betaine solution, 1 µl of 0.05 mol/L ethylenediaminetetraacetic acid disodium salt solution, and 8 µl of water in 96 well plate. Next, droplets were made on the plate containing the mixture using the QX200 AutoDG Droplet Digital PCR system (Bio-Rad). Droplets were subjected to PCR and then analyzed. Sample dilution was not done prior to PCR reaction. The PCR cycling conditions were 95 °C for 10 min, followed by 40 cycles of 95 °C for 30 s and 58 °C for 2 min, and 98 °C for 10 min. Data from the ddPCR experiments were analyzed using QuantaSoft analysis software version 1.7.4 (Bio-Rad). The limit of blank (LoB) was determined by analyzing negative controls. We used DNA extracted from cultured cells as a control (negative control: HEK293, positive control: TCCSUP). We had performed limit of detection (LoD) analysis. Three samples could not be analyzed because their LoD values were below the LoB value, but the other samples could be analyzed because their LoD values were above the LoB value. Primer specificity was assessed by analyzing above control DNAs to ensure there was no cross-reactivity.

### *MDM2* and *ERBB2* copy number analysis

The copy numbers of *MDM*2 and *ERBB2* were measured using TaqMan Copy Number Assays (Thermo Fisher Scientific) and StepOnePlus qPCR^®^ (Thermo Fisher Scientific). cfDNA samples were mixed with a TaqMan Copy Number Assay for *MDM2* (Hs02970282) or *ERBB2* (Hs02428732), a TaqMan Copy Number Reference Assay for RNase P (4403326), and a Master Mix. Each gene was amplified using StepOnePlus qPCR. 1 µl of purified cfDNA was used for each reaction. The PCR cycling conditions were 95 °C for 10 min, followed by 40 cycles at 95 °C for 15 s. The copy numbers of *MDM2* and *ERBB2* were calculated using RNase P as an internal control and CopyCaller software version 2 (Thermo Fisher Scientific).

### Statistical analysis

Using imaging, patients were divided into the partial response (PR), stable disease (SD), and progressive disease (PD) groups before and after drug therapy, and the association with cfDNA levels between the groups was statistically analyzed using JMP^®^ software. Statistical analysis was performed with a t-test for the two corresponding groups. Differences were considered statistically significant at p < 0.05.

### Supplementary Information

Supplementary Information.

## Data Availability

The datasets used and analyzed in the current study are available from the corresponding author upon reasonable request.
