# Author Correction: Screening for a practical method to monitor the status of patients with metastatic bladder cancer at the circulating cell-gene level

**DOI:** 10.1038/s41598-023-49374-w

**Published:** 2023-12-15

**Authors:** Ryota Ogura, Saya Ito, Takashi Ueda, Yusuke Gabata, Satoshi Sako, Yuta Inoue, Takeshi Yamada, Hirotaka Konishi, Atsuko Fujihara, Osamu Ukimura

**Affiliations:** 1 Department of Urology, Graduate School of Medical Science, Kyoto Prefectural University of Medicine, Kyoto, Kyoto 602-8566 Japan; 2 Division of Digestive Surgery, Department of Surgery, Kyoto Prefectural University of Medicine, Kyoto, Kyoto Japan

Correction to: *Scientific Reports* 10.1038/s41598-023-46977-1, published online 09 November 2023

The original version of this Article contained an error in the Results, under the subheading ‘Quantification of cfDNA in patients with mBC is a possible biomarker to monitor therapeutic efficacy’,

“In the PD group, although cfDNA levels tended to increase in the chemotherapy group, no significant difference was observed (Fig. 2) (p < 0.05).”

now reads:

“In the PD group, although cfDNA levels tended to increase in the chemotherapy group, no significant difference was observed (Fig. 2) (p=0.0605).”

The original Article has been corrected.

