# Supplementary material for: Screening for a practical method to monitor the status of patients with metastatic bladder cancer at the circulating cell-gene level
Source: Sci Rep. 2023 Nov 9;13:19517. doi: 10.1038/s41598-023-46977-1 (PMC10636091; doi:10.1038/s41598-023-46977-1)

**Screening for a practical method to monitor the status of patients with metastatic bladder cancer at the circulating cell-gene level**

Ryota Ogura ^1†^, MD; Saya Ito^1^, PhD; Takashi Ueda^1^**^*^**, MD, PhD; Yusuke Gabata^1^, MD; Satoshi Sako^1^, MD; Yuta Inoue^1^, MD, PhD; Takeshi Yamada^1^, MD, PhD; Hirotaka Konishi^2^, MD, PhD; Atsuko Fujihara^1^, MD, PhD; Osamu Ukimura^1^, MD, PhD

^1^ Department of Urology, Kyoto Prefectural University of Medicine, Kyoto-City, Kyoto, Japan

^2^ Division of Digestive Surgery, Department of Surgery, Kyoto Prefectural University of Medicine, Kyoto-City, Kyoto, Japan

^*^ Corresponding author. Takashi Ueda, MD PhD

Address: Department of Urology, Graduate School of Medical Science, Kyoto Prefectural University of Medicine, Kyoto-City, Kyoto 602-8566, Japan

FAX: +81 75 251 5598

TEL: +81 75 251 5595

E-mail: [t-ueda@koto.kpu-m.ac.jp](mailto:t-ueda@koto.kpu-m.ac.jp)

**Supplementary Information**

**Table S1.** **Cases with positive TERTC228T mutation**

This is a case with a positive TERTC228T mutation.

Abbreviations:Pre, before treatment; Post, after treatment; -, negative

**Table S2.** **Characteristics of healthy people**

One patient had a history of Basedow's disease (currently under control with　thiamazole), and the other 9 were healthy subjects without any particular history. The median normal cfDNA concentration was 3.78 ng/ml.

Abbreviations:F, female; M, male

**Figure S1. CT images and genetic mutations in the cfDNA of patients in the SD group.** Representative data from case 2 are shown. There were no predominant changes in cfDNA levels, genetic variants in *TERT* (C228T), and *ERBB2* copy numbers.

**Figure S2. Summary of this study.** The observed decrease in cfDNA levels after treatment likely reflects a PR during therapeutic evaluation. The increase in ctDNA levels shown by the C228T mutation in the *TERT* gene and/or the increase in *ERBB2* gene copy number post-treatment is defined as PD.

Abbreviations:↑, increased; ↓, decreased; +, positive; -, negative


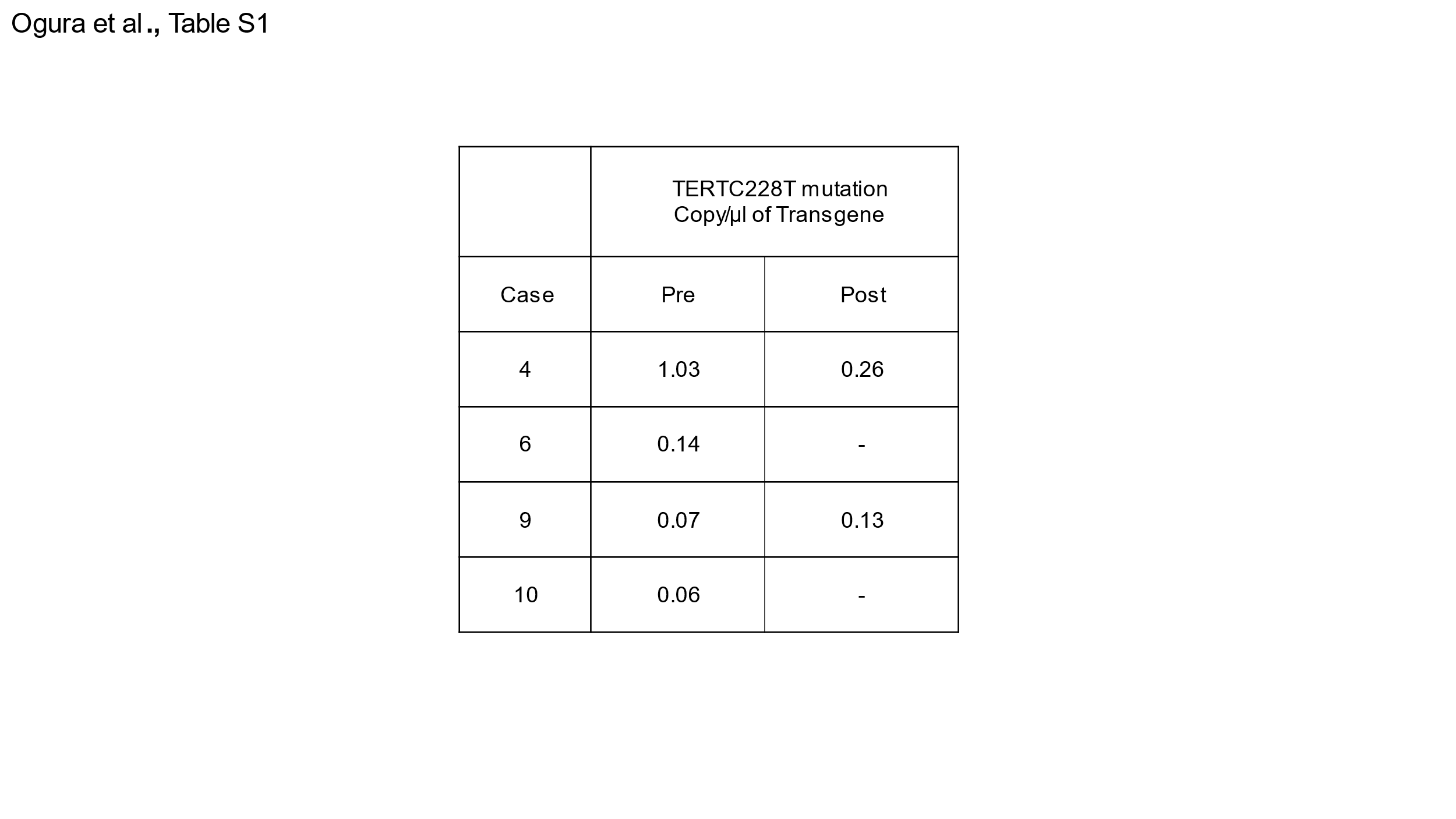


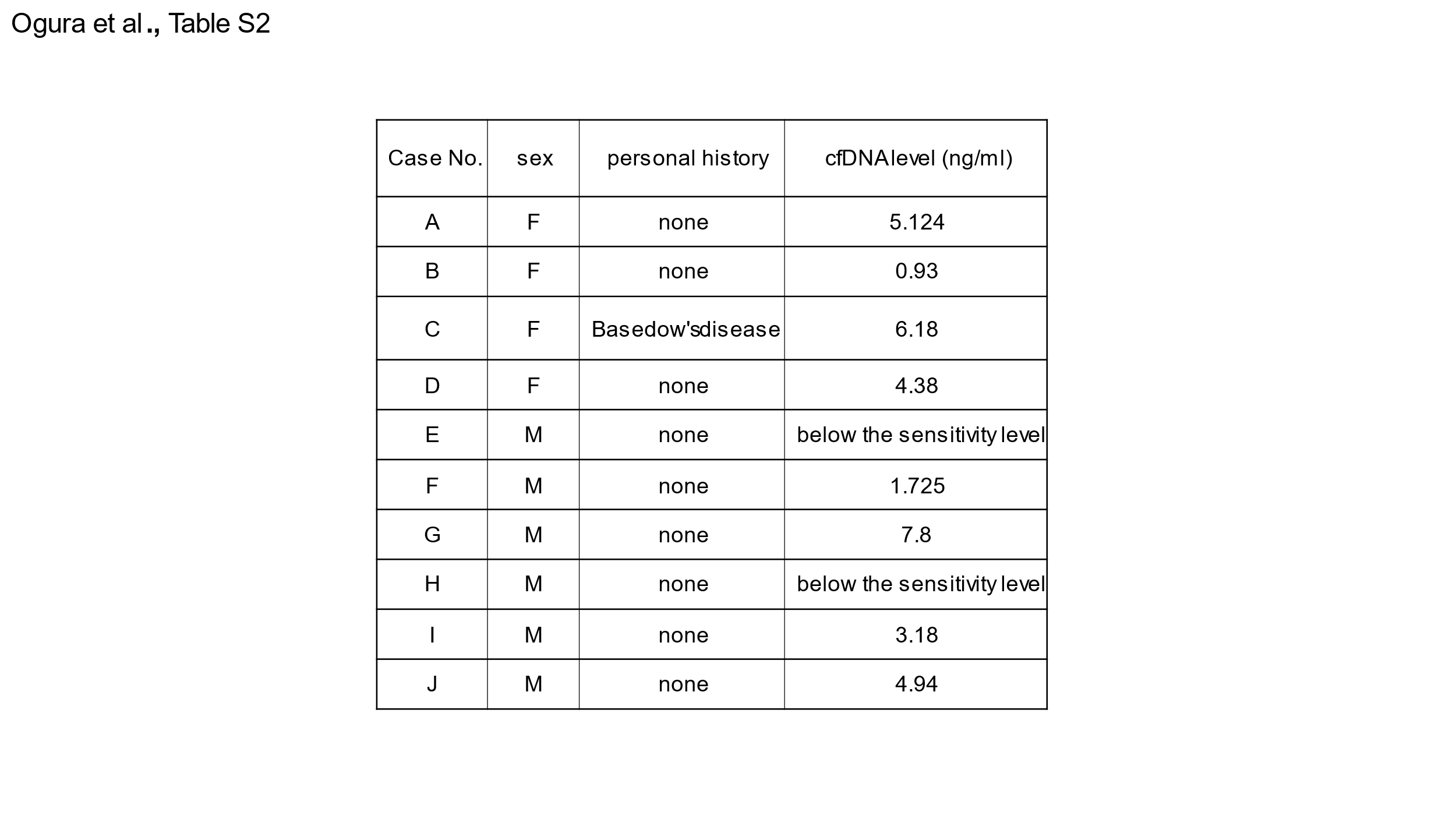


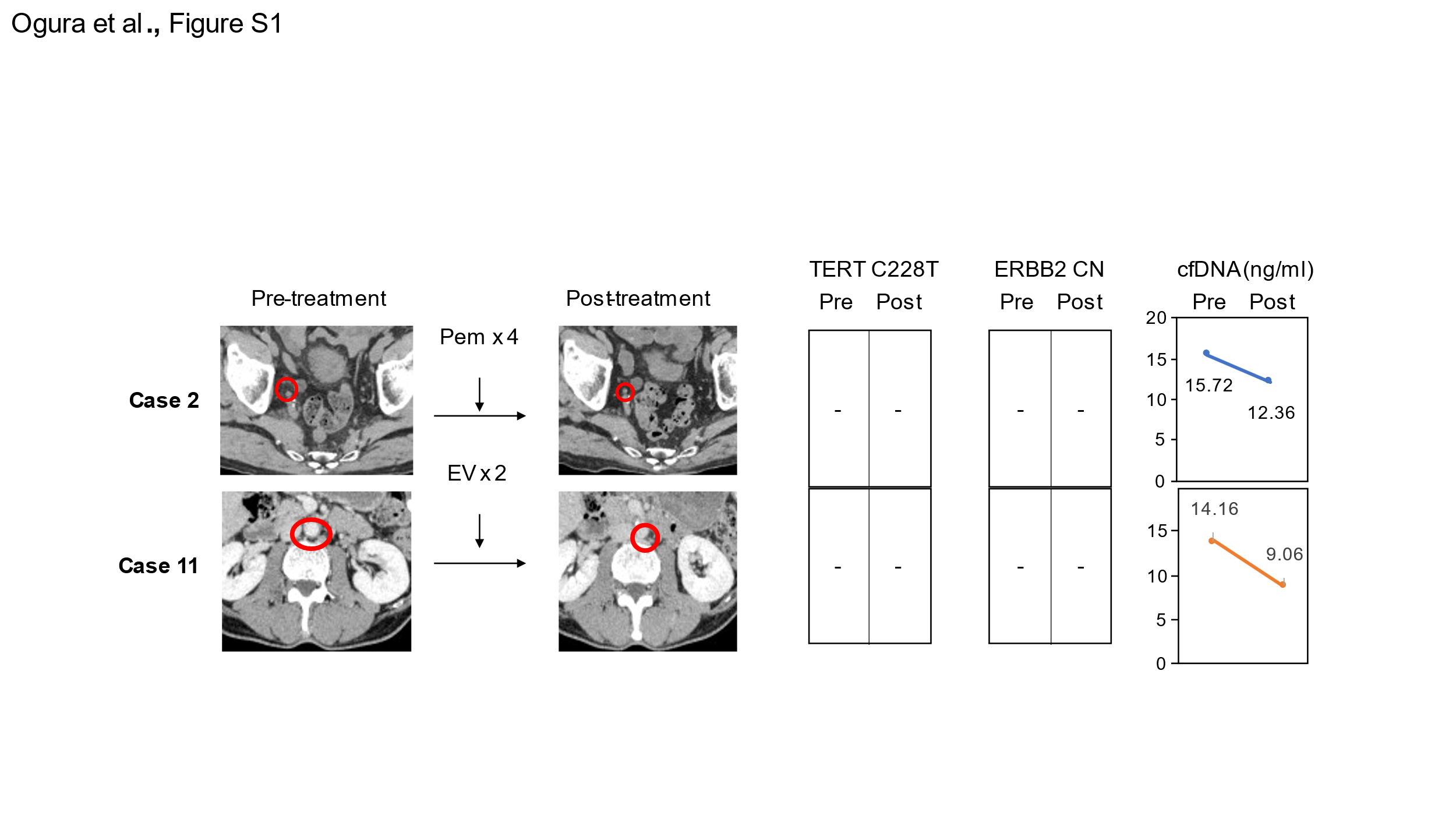


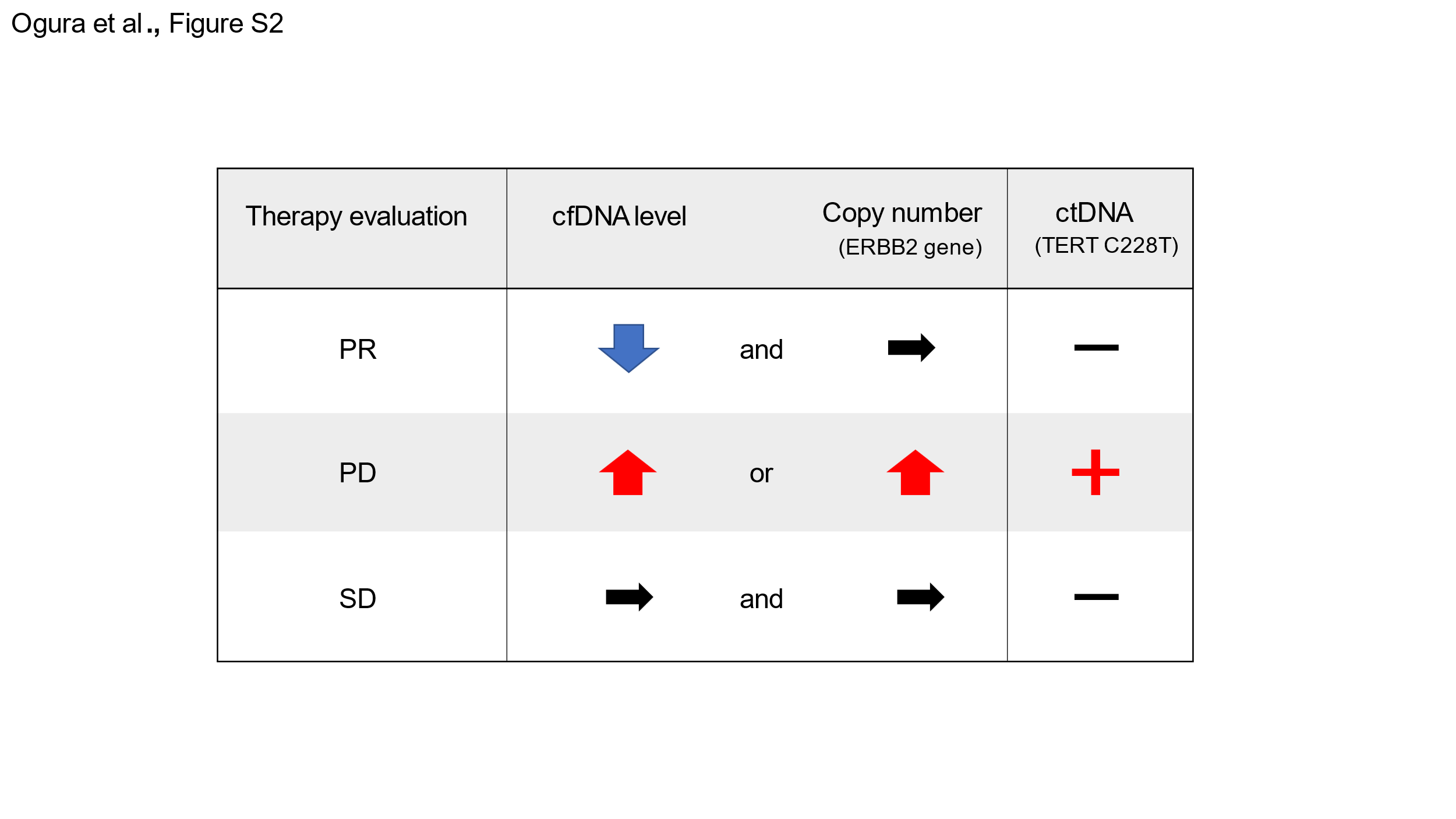

Supplement: Supplementary file 1 — Supplementary Information. [file 41598_2023_46977_MOESM1_ESM.docx]
